# Lactacystin-Induced Model of Hypertension in Rats: Effects of Melatonin and Captopril

**DOI:** 10.3390/ijms18081612

**Published:** 2017-07-25

**Authors:** Fedor Simko, Olga Pechanova, Kristina Repova, Silvia Aziriova, Kristina Krajcirovicova, Peter Celec, Lubomira Tothova, Stanislava Vrankova, Lucia Balazova, Stefan Zorad, Michaela Adamcova

**Affiliations:** 1 Institute of Pathophysiology, Faculty of Medicine, Comenius University, Sasinkova 4, 81108 Bratislava, Slovakia; repova.k@gmail.com (K.R.); silvia.aziriova@gmail.com (S.A.); krikratina@gmail.com (K.K.); petercelec@gmail.com (P.C.); 2 3rd Clinic of Internal Medicine, Faculty of Medicine, Comenius University, 83305 Bratislava, Slovakia; 3 Institute of Experimental Endocrinology, Biomedical Research Center, Slovak Academy of Sciences, 84505 Bratislava, Slovakia; balazova.luc@gmail.com (L.B.); Stefan.Zorad@savba.sk (S.Z.); 4 Institute of Normal and Pathological Physiology, Slovak Academy of Sciences, 81371 Bratislava, Slovakia; olga.pechanova@savba.sk (O.P.); stanislava.vrankova@savba.sk (S.V.); 5 Institute of Molecular Biomedicine, Faculty of Medicine, Comenius University, 81108 Bratislava, Slovakia; tothova.lubomira@gmail.com; 6 Department of Physiology, Faculty of Medicine, Charles University, 50003 Hradec Kralove, Czech Republic; adamcova@lfhk.cuni.cz

**Keywords:** lactacystin, hypertension, fibrosis, captopril, melatonin, remodelling

## Abstract

Lactacystin is a proteasome inhibitor that interferes with several factors involved in heart remodelling. The aim of this study was to investigate whether the chronic administration of lactacystin induces hypertension and heart remodelling and whether these changes can be modified by captopril or melatonin. In addition, the lactacystin-model was compared with N^G^-nitro-l-arginine-methyl ester (L-NAME)- and continuous light-induced hypertension. Six groups of three-month-old male Wistar rats (11 per group) were treated for six weeks as follows: control (vehicle), L-NAME (40 mg/kg/day), continuous light (24 h/day), lactacystin (5 mg/kg/day) alone, and lactacystin with captopril (100 mg/kg/day), or melatonin (10 mg/kg/day). Lactacystin treatment increased systolic blood pressure (SBP) and induced fibrosis of the left ventricle (LV), as observed in L-NAME-hypertension and continuous light-hypertension. LV weight and the cross-sectional area of the aorta were increased only in L-NAME-induced hypertension. The level of oxidative load was preserved or reduced in all three models of hypertension. Nitric oxide synthase (NOS) activity in the LV and kidney was unchanged in the lactacystin group. Nuclear factor-kappa B (NF-κB) protein expression in the LV was increased in all treated groups in the cytoplasm, however, in neither group in the nucleus. Although melatonin had no effect on SBP, only this indolamine (but not captopril) reduced the concentration of insoluble and total collagen in the LV and stimulated the NO-pathway in the lactacystin group. We conclude that chronic administration of lactacystin represents a novel model of hypertension with collagenous rebuilding of the LV, convenient for testing antihypertensive drugs or agents exerting a cardiovascular benefit beyond blood pressure reduction.

## 1. Introduction

Hypertensive heart disease is a serious consequence of hypertension. Left ventricular hypertrophy (LVH) is a compensatory response to chronically-increased haemodynamic load which enhances the heart performance without substantially increasing wall tension and energy consumption. However, since hypertrophied myocardium differs from normal myocardium, LVH represents an independent cardiovascular risk [1,2,3,4]. Thus, there is a continuous effort to identify substances that prevent or reverse LVH [5,6,7,8]. However, besides the nature of the drug per se, the effectiveness of anti-remodelling protection depends on the type and severity of the overload and the concomitant neurohumoral alterations that modify myocyte and fibrocyte activity. Bearing this in mind, it is essential to test potentially protective strategies in different models of pathological myocardial growth [9,10,11].

The ubiquitin-proteasome system degrades many cytosolic, nuclear and myofibrillar proteins [12]. Lactacystin is a proteasome inhibitor that interferes with the synthesis and degradation of several proteins involved in cardiovascular organ remodelling such as nuclear factor-kappa B (NF-κB), a nuclear transcriptional factor [13], tyrosine hydroxylase, the rate limiting enzyme in catecholamine biosynthesis [14], cyclooxygenase-2, a marker of inflammation [15], and the sarcomeric myosin heavy chain [16].

Due to the complex effect of lactacystin on several factors differently involved in the hypertrophic growth of the heart and vessels, the aim of this study was to determine whether the chronic treatment of Wistar rats with lactacystin is able to induce hypertension and the pathological remodelling of the heart and aorta. Moreover, we sought to determine whether captopril, the classical angiotensin converting enzyme (ACE)-inhibitor with antihypertensive and anti-remodelling effects or melatonin, which was previously shown to have an anti-remodelling nature [17,18,19,20,21,22], supposedly related to its antioxidant [23,24,25], nitric oxide (NO)-bioavailability enhancing [26,27,28] and chronobiologic actions [29,30,31], could modify the potential alterations induced by lactacystin. Furthermore, we compared the lactacystin-model with the well-established N^G^-nitro-l-arginine-methyl ester (L-NAME)- and continuous light-induced hypertension in rats.

## 2. Results

### 2.1. Cardiovascular Parameters

**Figure 1:**
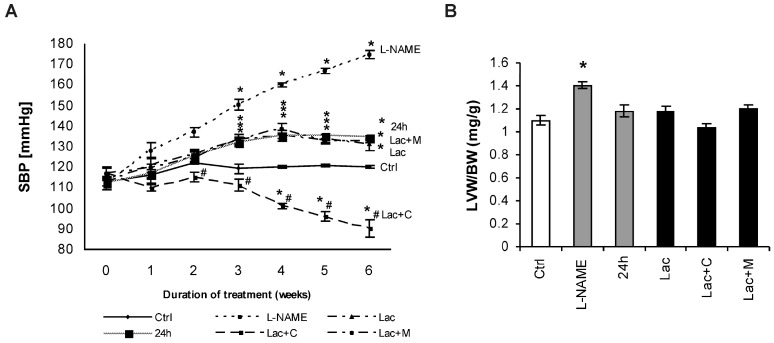
Systolic blood pressure (SBP) (**A**) and relative left ventricular weight (LVW/BW) (**B**) in the control group (Ctrl), L-NAME (L-NAME)-, continuous 24 h/day light (24 h)-, lactacystin (Lac)-induced hypertension, and in lactacystin-hypertension influenced by captopril (Lac+C) or melatonin (Lac+M). * *p* < 0.05 vs. Ctrl, # *p* < 0.05 vs. Lac.

After six weeks of treatment, systolic blood pressure (SBP) was 120 ± 0.48 mmHg in the control and was enhanced to 174 ± 2.17 mmHg, 134.5 ± 1.28 mmHg, and 131.2 ± 3.45 mmHg in the L-NAME, 24 h and lactacystin (Lac) groups, respectively (enhancement by 45%, 12%, and 9% respectively, *p* < 0.05 for all). SBP was decreased significantly (*p* < 0.05) by captopril (31%), while melatonin had no effect on SBP (Figure 1A). The left ventricle weight/body weight (LVW/BW) ratio after six weeks of treatment was 1.10 ± 0.04 mg/g in the control and was only increased by L-NAME-treatment (by 28%, *p* < 0.05). Captopril slightly reduced the LVW/BW ratio (by 8%) compared to Lac group (Figure 1B).

### 2.2. Morphometry of the Aorta

**Figure 2:**
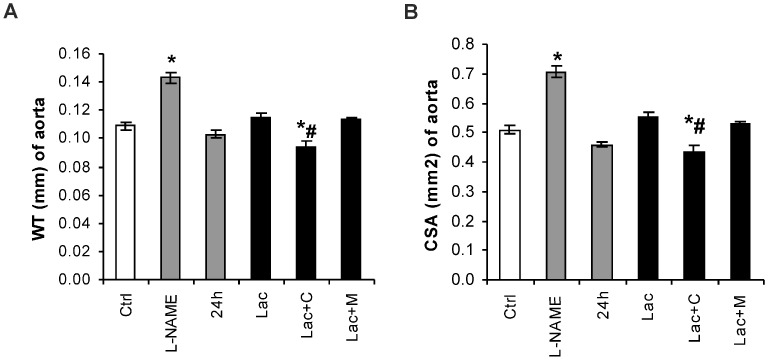
Aortic thickness (WT) (**A**) and cross sectional area (CSA) (**B**) in the control group (Ctrl), L-NAME (L-NAME)-, continuous 24 h/day light (24 h)-, lactacystin (Lac)-induced hypertension, and in lactacystin-hypertension influenced by captopril (Lac+C) or melatonin (Lac+M). * *p* < 0.05 vs. Ctrl, # *p* < 0.05 vs. Lac.

The wall thickness (WT) was 0.109 ± 0.003 mm in the control and was increased by L-NAME treatment by 31% (*p* < 0.05) (Figure 2A). The cross-sectional area of the aorta was 0.510 ± 0.013 mm^2^ in the control and was enhanced by L-NAME treatment by 38% (*p* < 0.05). In the Lac group, captopril reduced WT by 18% (*p* < 0.05) and the cross-sectional area (CSA) by 22% (*p* < 0.05) (Figure 2B).

### 2.3. Hydroxyproline in Soluble and Insoluble Collagenous Fraction in the Left Ventricle (LV)

**Figure 3:**
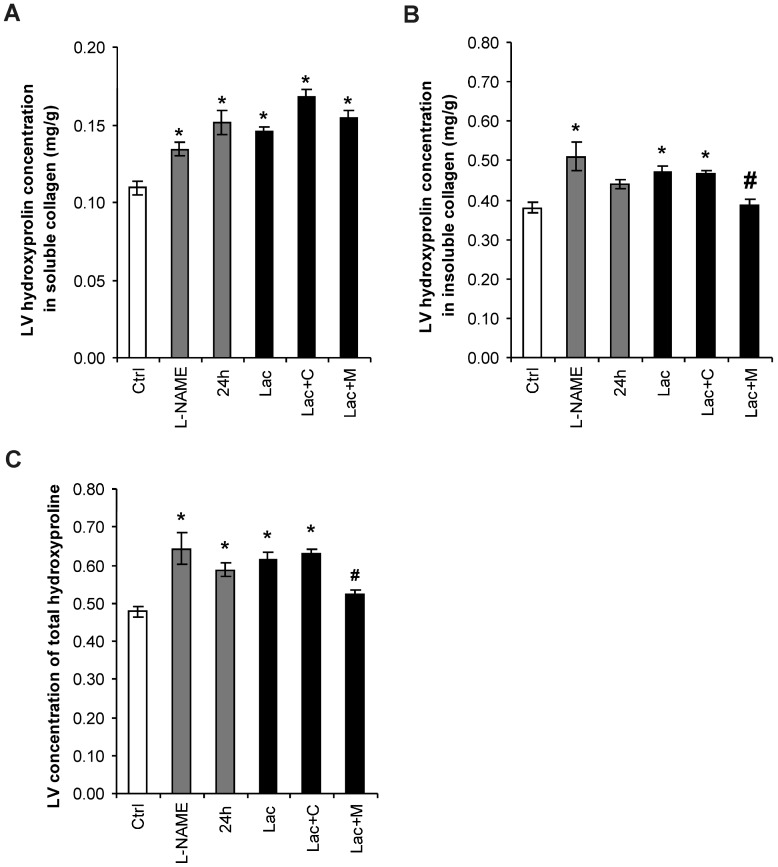
Hydroxyproline concentration in the LV in soluble (**A**) and insoluble (**B**) collagen, and total hydroxyproline (**C**) in the control group (Ctrl), L-NAME (L-NAME)-, continuous 24 h/day light (24 h)-, lactacystin (Lac)-induced hypertension, and in lactacystin-hypertension influenced by captopril (Lac+C) or melatonin (Lac+M). * *p* < 0.05 vs. Ctrl, # *p* < 0.05 vs. Lac.

Hydroxyproline concentration in the soluble collagenous proteins was 0.11 ± 0.004 mg/g in the control group and was increased in L-NAME, 24 h and Lac by 20%, 36% and 37%, respectively (all *p* < 0.05). Neither captopril nor melatonin produced changes in the Lac group. Hydroxyproline concentration in the insoluble collagenous proteins was 0.38 ± 0.015 mg/g in the control group and was increased in L-NAME, 24 h, and Lac by 34%, 16%, and 24%, respectively (all *p* < 0.05). Melatonin reduced the level of insoluble collagen by 17% (*p* < 0.05) in the Lac group. The sum of hydroxyproline in soluble and insoluble fractions was 0.48 ± 0.014 mg/g in the control group and was increased in L-NAME, 24 h, and Lac by 33%, 23%, and 29%, respectively (all *p* < 0.05). Melatonin reduced total hydroxyproline by 16% (*p* < 0.05) in the Lac group (Figure 3).

### 2.4. NO-Synthase (NOS) Activity in the LV and Kidney

**Figure 4:**
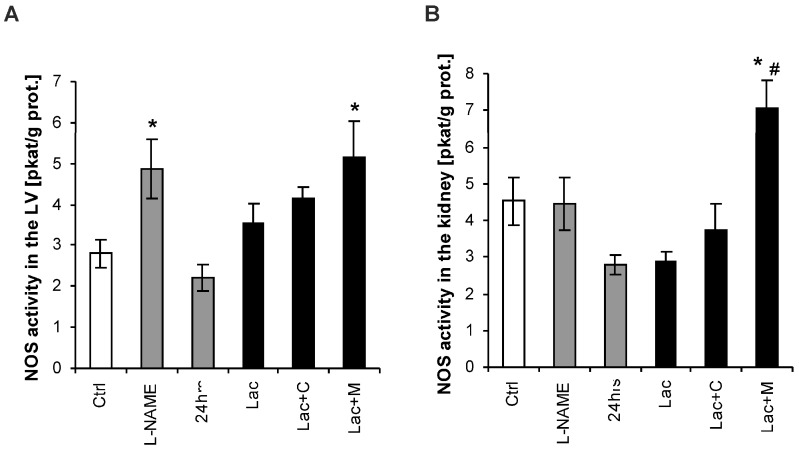
Nitric oxide synthase (NOS) activity in the heart (**A**) and kidney (**B**) in the control group (Ctrl), L-NAME (L-NAME)-, continuous 24 h/day light (24 h)-, lactacystin (Lac)-induced hypertension and in lactacystin-hypertension influenced by captopril (Lac+C) or melatonin (Lac+M). * *p* < 0.05 vs. Ctrl, # *p* < 0.05 vs. Lac.

NOS activity in the LV was 2.92 ± 0.41 pkat/g/protein in the control group and increased by 69% (*p* < 0.05) in the L-NAME group. NOS activity was 3.78 ± 0.28 pkat/g protein in the Lac group and was increased by 37% (*p* < 0.05) by melatonin (Figure 4A).

NOS activity in the kidney was 4.52 ± 0.30 pkat/g protein in the control group and was not changed in either model of hypertension. NOS activity was 3.58 ± 0.08 pkat/g protein in Lac and was increased by 95% (*p* < 0.05) by melatonin (Figure 4B).

### 2.5. Oxidative Stress Parameters

**Figure 5:**
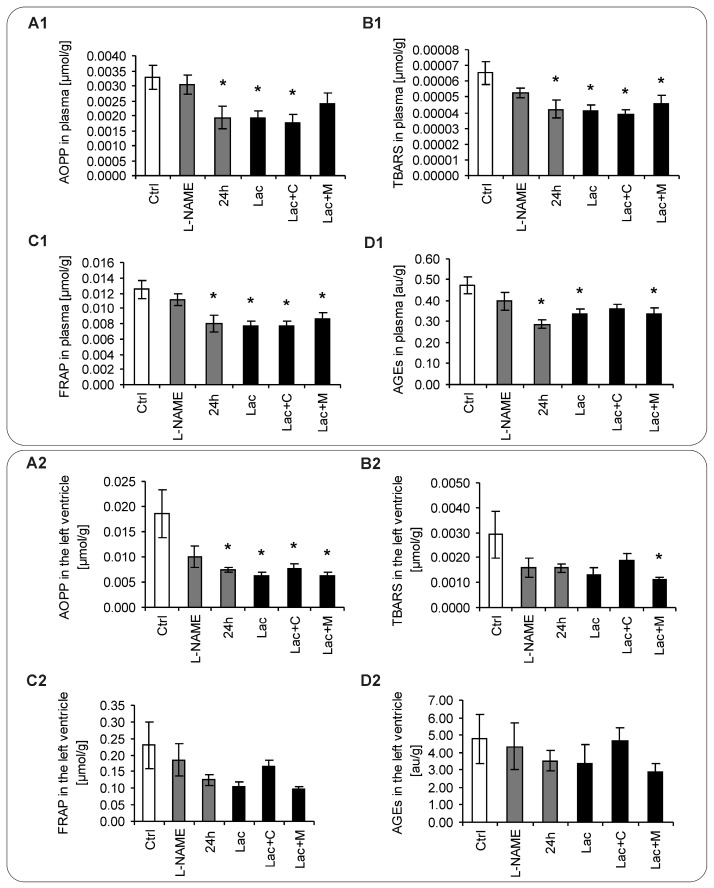

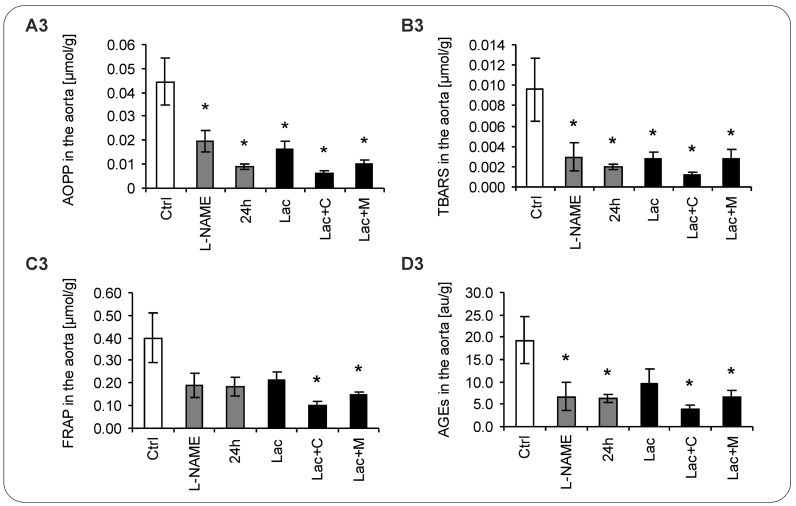
The parameters of oxidative status in plasma (**A1**–**D1**), left ventricle (LV) (**A2**–**D2**) and aorta (**A3**–**D3**) in the control group (Ctrl), L-NAME (L-NAME)-, continuous 24 h/day light (24 h)-, lactacystin (Lac)-induced hypertension and in lactacystin-hypertension influenced by captopril (Lac+C) or melatonin (Lac+M). * *p* < 0.05 vs. Ctrl.

After six weeks of treatment, the advanced oxidation protein products (AOPP) in plasma was 0.0032 ± 0.00041 μmol/g in the control group, and declined significantly in 24 h, Lac, Lac+C by 41%, 42%, 46% (*p* < 0.05), respectively and numerically in Lac+M by 27% (Figure 5A1). The plasma level of thiobarbituric acid-reacting substances (TBARS) was 0.0000654 ± 0.71 μmol/g in the control group and declined in 24 h, Lac, Lac+C and Lac+M by 35%, 37%, 41%, 30% (all *p* < 0.05), respectively (Figure 5B1). The concentration of ferric reducing antioxidant power (FRAP) was 0.013 ± 0.0011 μmol/g in the control group and declined in 24 h, Lac, Lac+C, and Lac+M by 36%, 39%, 37%, 15% (*p* < 0.05), respectively (Figure 5C1). The plasmatic advanced glycation end-products (AGEs) were 0.474 ± 038 μmol/g in the control group and declined significantly in 24 h, Lac, and Lac+M by 39%, 29%, 29% (*p* < 0.05), respectively, and numerically in Lac+C by 24% (Figure 5D1).

After six weeks of treatment, the AOPP in the LV was 0.0185 ± 0.0048 μmol/g in the control group, and declined significantly in 24 h, Lac, Lac+C, and Lac+M by 60%, 66%, 58%, 65 (*p* < 0.05), respectively, and numerically in L-NAME by 46% (Figure 5A2). The level of TBARS in the LV was 0.00292 ± 0.00093 μmol/g in the control group, and declined significantly in Lac+M by 62% (*p* < 0.05) and numerically in L-NAME, 24 h, Lac, Lac+C by 46%, 46%, 55%, 35%, respectively (Figure 5B2). The concentration of FRAP in the LV was 0.229 ± 0.072 μmol/g in the control group and was not changed significantly in either group. (Figure 5C2). The level of AGEs in the LV was 4.773 ± 1.389 μmol/g in the control group and was not changed significantly in either group (Figure 5D2).

After six weeks of treatment, the AOPP in the aorta was 0.0444 ± 0.0097 μmol/g in the control group, and declined significantly in L-NAME, 24 h, Lac, Lac+C by 56%, 80%, 63%, 86%, 76% (*p* < 0.05), respectively (Figure 5A3). The aortic level of TBARS was 0.0096 ± 0.0031 μmol/g in the control group and declined in L-NAME, 24 h, Lac, Lac+C, and Lac+M by 69%, 79%, 70%, 87%, 70% (*p* < 0.05), respectively (Figure 5B3). The aortic concentration of FRAP was 0.399 ± 0.110 μmol/g in the control group and declined in Lac+C and Lac+M significantly by 51% and 30%, respectively (*p* < 0.05) and in L-NAME, 24 h, and Lac numerically by 52%, 53%, 46%, respectively (Figure 5C3). The aortic AGEs were 19.24 ± 5.24 μmol/g in the control group and declined significantly in L-NAME, 24 h, Lac+C and Lac+M by 65%, 67%, 79%, 65%, respectively (*p* < 0.05), and in Lac numerically by 49% (Figure 5D3).

### 2.6. Nuclear Factor-Kappa B (NF-κB) Expression

**Figure 6:**
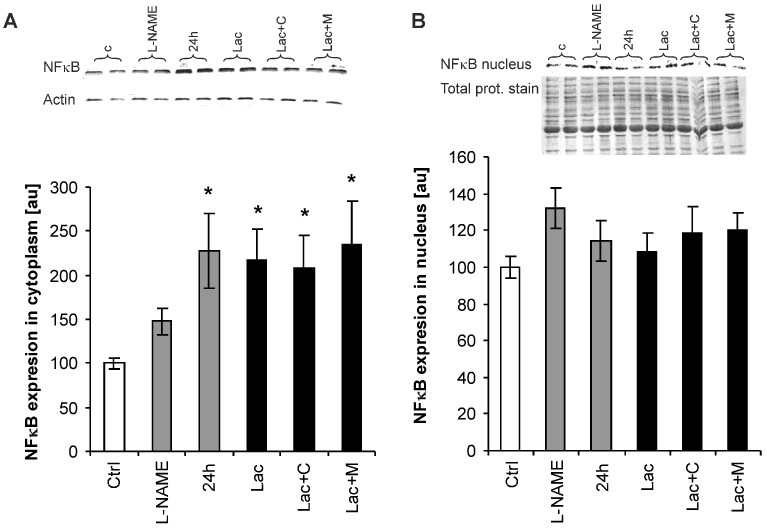
Cytoplasmatic (**A**) and nuclear (**B**) left ventricular NF-κB expression in the control group (Ctrl), L-NAME (L-NAME)-, continuous 24 h/day light (24 h)-, lactacystin (Lac)-induced hypertension, and in lactacystin-hypertension influenced by captopril (Lac+C) or melatonin (Lac+M). * *p* < 0.05 vs. Ctrl.

In the cytoplasm, NF-κB expressed as a percentage of the control group increased in L-NAME, 24 h and Lac by 148%, 216%, and 228% (*p* < 0.05), respectively. Neither captopril nor melatonin produced changes in the Lac group (maintained enhancement by 208% and 235% compared to controls, *p* < 0.05) (Figure 6A). In the nucleus, the expression of NF-κB as a percentage of the control group remained unchanged in all groups (Figure 6B).

## 3. Discussion

A slight but significant SBP increase was observed in rats treated with lactacystin in this experiment. This mild SBP enhancement was similar to that of continuous light hypertension, but much lower compared to L-NAME-hypertension, where SBP increased by about 30%. It corresponded with the changes in LV mass. While in the lactacystin-model (similarly to continuous light hypertension) no LVH developed after six weeks of treatment, L-NAME-hypertension caused significant LVH development as well as hypertrophy of the aorta. This is associated with the fact that the extent of haemodynamic overload is closely connected to hypertrophic myocardial growth [3,4,32]. On the other hand, fibrotic remodelling in terms of enhancement of soluble, insoluble, and total collagen level in the LV observed in lactacystin-treated animals was analogical to fibrotic alterations present in continuous light- and L-NAME-induced hypertension. It supports the opinion held by several authors that the interstitial matrix is predominantly modified by factors other than hemodynamic load per se, such as neurohumoral activation.

The mechanisms of organ rebuilding in hypertension may be related to oxidative stress or l-arginine-NO pathways. However, no enhancement of oxidative stress was observed in the plasma, LV or aorta in either model. In general, the parameters of oxidative load even decreased. Indeed, free radicals may act not just as damaging factors that stimulate pathologic rebuilding of the heart and vessels but also as signalling or protective factors [33], or molecules indicating or administering adaptive alterations in different periods of hypertrophic growth [34]. Oxidative stress associated with hypertension seems to be the consequence rather than the cause of long-lasting hypertension [35]. Large clinical studies have not recorded any reduction in blood pressure or cardiovascular events after long-term treatment with variable antioxidants [36]. In fact, it has never been proved that patients who presented with negative results actually had increased oxidative stress [37]. The shortage of free radicals has even been hypothesized as an etiologic factor in type 2 diabetes [38]. In agreement with the data of our experiment it was previously shown that the inhibition of proteasome by lactacystin may result in decreasing the level of oxidative stress [39]. Thus, hypertension is not necessarily associated with the overproduction of free radicals. Their level may depend on the period of hypertension and longevity of treatment [34].

The NO molecule is known to have both vasodilating and anti-proliferative properties [40]. However, NOS activity remained unchanged in lactacystin-induced hypertension (similar to that in continuous light-hypertension) and was increased in L-NAME-hypertension in the LV. Thus, NO-deficit does not seem to be a substantial factor in the development of fibrosis in lactacystin-induced hypertension.

Interestingly, captopril prominently reduced systolic blood pressure, while melatonin had no significant effect. However, captopril did not influence fibrosis, while melatonin reduced the hydroxyproline concentration in the insoluble and total collagen of the LV. This fibrosis-reducing effect of melatonin was consistently observed in several previous experiments with spontaneously hypertensive rats (SHR) [20], L-NAME-induced hypertension [41], in continuous light [21] and continuous light+L-NAME-induced hypertension [42], as well as in isopenaline induced heart failure [43]. Importantly, melatonin predominantly reduced insoluble ross-linked (matured) collagen, which is considered to be responsible for increased stiffness of the LV with diastolic dysfunction [21,43]. Since the quality and not the quantity of the hypertrophied myocardium is the decisive factor of the negative cardiovascular prognosis [44], the anti-fibrotic potential of melatonin may be of utmost benefit for patients with hypertensive heart disease or heart failure. Indeed, the reduction of the insoluble collagen level in isoprenaline-induced heart failure in rats was associated with decreased mortality [43]. The inability of melatonin to reduce SBP in this experiment indicates that the nature of melatonin to act against insoluble collagen accumulation is independent from the effect on haemodynamic load. The previous data indicating melatonin’s sympatholytic effect and interference with the renin-angiotensin system [45] suggest that the concise anti-fibrotic effect of melatonin in the heart is potentially determined by the attenuation of undesirable neurohumoral activation.

NF-κB is a ubiquitous inducible transcription factor [46], which seems to play a substantial role in hypertrophic myocardial growth [47]. There are several indications that NF-κB activation exerts unfavourable, pathological myocardial growth supporting actions. The NF-κB activation increased cardiac remodelling and dysfunction following myocardial infarction [48], the development of LVH induced by angiotensin II [49], and by increased afterload due to thoracic aorta banding [50]. Moreover, the reversal of cardiac hypertrophy was observed after the inhibition of NF-κB signalling by using a gene knockdown approach [46]. On the other hand, some data suggest that NF-κB may have a protective rather than a deleterious effect on a haemodynamically-overloaded heart. Although NF-κB transgenic inhibition attenuated LVH induced by aortic-constriction, the progression of LVH to maladaptive LV remodelling was not inhibited, which indicate that NF-κB is needed for adaptive cardiac hypertrophy [51]. Interestingly, melatonin was shown to inhibit the activity of NF-κB in a number pathologic states such as neurodegenerative and neurotoxic disturbances [52,53], in oncologic diseases [54,55,56], or in aging heart [57], potentially by increasing concentration of NF-κB inhibitor (IκBα) [58,59].

In this experiment, NF-κB expression in the cytoplasm was increased in all groups with hypertension including those with captopril and melatonin. However, after activation in the cytoplasm, NF-κB is translocated into the nucleus with the subsequent stimulation of the expression of various genes including those that participate in cardiac remodelling. Therefore, it is suggested that the nuclear fraction of NF-κB and not the cytoplasmatic fraction is decisive for transcriptional induction of particular genes [47]. NF-κB expression in the nucleus remained unchanged in the all three models of hypertension. Furthermore, it was also not modified by captopril or melatonin. It may be supposed that haemodynamic and proliferative effects of lactacystin were not mediated by NF-κB. However, the consideration regarding the role of NF-κB in pathological hypertrophic growth is more complex than previously supposed. Neurohumoral stimulators such as angiotensin II [60] activates the redox sensitive NF-κB factor, which is critical for initiating the complex inflammatory response involving a number of pro-inflammatory and pro-fibrotic cytokines, chemokines, cell adhesion molecules, and variable growth factors both in the heart and vasculature [61,62]. On the other hand, NF-κB stimulates also endothelial NO production with antioxidant, anti-inflammatory, and anti-proliferative action, potentially counterbalancing the undesirable effect of pathologic growth stimulating factors [63,64]. Thus, to elucidate the mechanisms of pathologic myocardial growth the mutual interplay between neurohumoral activators, oxidative stress, NF-κB, and its targeting molecules exerting either pro-proliferative or anti-remodeling effects should be investigated.

To the best of our knowledge, we have shown for the first time that the chronic administration of lactacystin induces a slight but significant SBP increase and the fibrotic remodelling of the LV. Since the nuclear fraction of NF-κB remained unchanged, it seems that the haemodynamic and proliferative effects of lactacystin were not triggered through the influence on NF-κB. Although the mechanism of lactacystin effects remain unclear, it does not seem to be determined by a deficit in NO-production. Although captopril prominently reduced SBP, it did not influence myocardial fibrosis. On the other hand, melatonin does not prevent hypertension development, but reduces the level of insoluble and total collagen which was associated with the enhancement of NOS activity in the LV and kidney. Thus, the protective action of melatonin might have been determined by the anti-proliferative effect of NO.

## 4. Experimental Procedures

### 4.1. Animals and Treatment

All experimental procedures of the project No 2017/08-221 were carried out in accordance with the Guide for the Care and Use of Laboratory Animals published by the US National Institute of Health (NIH publication no 8523, revised 1985) and approved by an Ethics Committee for approval of animal experimental projects of the Institute of Pathophysiology, Faculty of Medicine, Comenius University in Bratislava on the 26 August 2008. Male adult (three-month-old) Wistar rats were randomly divided into six groups (*n* = 11 in each group): age-matched control (Wistar) rats (Cont), rats treated with L-NAME (L-NAME) (40 mg/kg/day), rats exposed to 24 h/day continuous light (24 h), rats treated with lactacystin (Lac) (5 mg/kg/day) alone or together with either captopril (100 mg/kg/day) (Egis Pharmaceuticals Ltd., Budapest, Hungary) (Lac+C), or melatonin (10 mg/kg/day) (Lac+M). Captopril, melatonin, L-NAME and lactacystin were dissolved in drinking water and their concentrations were adjusted to daily water consumption to ensure the correct dosage. Solutions containing melatonin were protected from light exposure. All rats were kept in individual cages at 22–24 °C and fed with a regular pellet diet ad libitum. SBP was measured each week by non-invasive tail-cuff plethysmography (Hugo-Sachs Elektronik, Freiburg, Germany). After six weeks, the rats were decapitated and the tissues and samples (LV, kidney, and blood) were collected. Weight of the heart (HW), left ventricle (LVW) and right ventricle (RVW) were determined and their relative weights (LVW/body weight and RVW/body weight ratio) were calculated. Samples of the left ventricle were frozen at −80 °C and later used for the determination of hydroxyproline, oxidative stress and NF-κB concentrations. In addition, blood samples were collected in EDTA tubes, centrifuged and the plasma was stored at −80 °C for the subsequent determination of oxidative stress parameters. Unless stated otherwise, all chemicals were purchased from Sigma Chemical Co. (Steinheim, Germany).

### 4.2. Morphometry of the Aorta

Formaldehyde fixed thoracic aorta samples were processed in paraffin, 5 μm thick sections were stained with haematoxylin and eosin and morphometric parameters were evaluated by light microscopy and a two-dimensional image analyser (Impor Pro; Kvant s.r.o., Bratislava, Slovakia). The wall thickness (WT) and the inner circumference in mm were measured and the cross-sectional area (CSA) in mm^2^ was calculated [65].

### 4.3. Determination of Hydroxyproline

The samples from the left ventricle were incrementally treated with different buffers as described previously [66,67]. The soluble collagenous proteins were isolated with a pepsin buffer with 0.5 mol/L CH_3_COOH. The insoluble collagenous proteins were isolated with 1.25 mol/L NaOH. The hydroxyproline concentration (a marker of fibrosis) was estimated in both collagenous fractions using spectrophotometry at 550 nm [68].

### 4.4. Assay of NO-Synthase (NOS) Activity

Total NOS activity was determined in crude LV and kidney tissue homogenates by measuring the formation of [3H]-l-arginine (Amersham International plc, Little Chalfont, UK), as described previously [69] with some modifications. The homogenates (10%, 50 μL) were incubated in the presence of NOS substrate (20 μmol/L [3H]-l-arginine with a specific activity 5 GBq/mmol, about 100,000 dpm/min) and NOS cofactors (30 nmol/L calmodulin, 1 mmol/L β-NADPH, 3 μmol/L tetrahydrobiopterine and 2 mmol/L Ca^2+^) in a total volume of 100 μL 50 nmol/L Tris-HCl (pH 7.4). After 10 min of incubation at 37 °C, the reaction was halted by adding 1 mL of 20 mmol/L HEPES buffer pH 5.5, containing 2 mmol/L EDTA, 2 mmol/L EGTA, and 1 mmol/L L-citrulline. Thereafter, the samples were centrifuged for 1 min at 4 °C (10,000× *g*). Supernatants were applied to 1 mL Dowex 50 WX-8 columns (Na^+^ form). L-[3H] citrulline eluated by 1 mL of water was measured by liquid scintillation counting. Finally, NOS activity was given as a picokatal per gram of protein.

### 4.5. Oxidative Load Measurement

Samples of plasma, LV and aorta were assayed for oxidative stress markers. Specific fluorescence (λex. = 370 nm, λem. = 440 nm) was measured in order to assess AGEs (advanced glycation end-products) as a marker of carbonyl stress. [70]. The calibration curve was constructed using advanced glycation endproduct-bovine serum albumin (AGE-BSA) standard according to [71]. The standard spectrophotometrical method was used to evaluate advanced oxidation protein products (AOPP) [72]. AOPP concentration was calculated on the basis of a chloramine T calibration curve with potassium iodide. Thiobarbituric acid-reacting substances (TBARS) of lipid peroxidation markers were measured according to Behuliak et al. [73]. TBARS contents were quantified based on a 1,1,3,3-tetramethoxypropane calibration curve. The ferric reducing ability of plasma or tissue homogenates (FRAP), as a measure of the antioxidant status in plasma or tissues was assessed [74]. The concentration of proteins in samples was estimated by a commercially available bicinchoninic acid assay. All measurements were performed using spectrofluorometer Saphire II (Tecan, Gradig, Vienna, Austria).

### 4.6. Western Blotting of NF-κB

Nuclear proteins were isolated using a high salt extraction protocol as described previously [75]. Protein concentration was determined by Bradford (Thermo Fisher Scientific, Waltham, MA, USA). Samples were subjected to SDS-PAGE (sodium dodecyl sulphate polyacrylamide gel electrophoresis). After blocking, blots were incubated overnight at 4 °C with primary antibody anti-NF-κB p65 (sc-372; Santa Cruz Biotechnology, Dallas, TX, USA). After membrane washing, the signal of fluorescently-labelled secondary antibodies (#5151 and #5257; Cell Signalling Technology, Danvers, MA, USA) was detected using an Odyssey infrared imager (LI-COR Biosciences, Lincoln, NE, USA). β-actin was used as endogenous loading control (#3700; Cell Signalling Technology, Danvers, MA, USA) for cytosolic fraction. The total protein stain with Coomassie Brilliant Blue was used to normalize the nuclear target protein expression [76]. Protein levels were quantified through the use of Odyssey IR imaging system software ver. 2.0 (LI-COR Bioscences, Lincoln, NE, USA).

### 4.7. Statistical Analysis

The results are expressed as mean ± S.E.M. A one-way, two-tailed analysis of variance (ANOVA) and the Bonferroni post-hoc test were used for statistical analysis. A nonparametric Kruskal-Wallis test and a Mann-Whitney test were used for the statistical analysis of NF-κB expression. Differences were considered significant at a *p*-value < 0.05.

## 5. Limitations

To reliably interpret the role of NF-κB in pathological hypertrophic growth, various methodological approaches such as investigation of the amount of NF-κB vs. its DNA-binding activity in the nuclear extract should be considered. Despite increased cytosolic NF-κB level in different models of hypertension in our study, there were no significant changes of NF-κB protein amount in nuclear extracts of heart in any model. Since nuclear localization is a prerequisite for transcription factor binding, we do not expect altered NF-κB DNA binding activity following hypertension in the presented models. Although several posttranslational modifications of NF-κB p65 subunits modulating its activity, such as phosphorylation or acetylation are known, these actions occur in cytoplasm prior to nuclear translocation. Importantly, nuclear phosphorylation of NF-κB was shown to determine target gene specificity, but not its binding activity [77,78]. Moreover, numerous studies found altered NF-κB binding activity only concomitantly with changes in nuclear NF-κB p65 protein amount [79,80]. Thus, it does not seem unreasonable to suppose that quantification of nuclear NF-κB protein could represent a reliable indicator of its activation.

The study might have provided more complex insights if the control group with captopril or melatonin would have been involved in the design. However, to increase the number of simultaneously-studied groups and animals was beyond our technical capacity. Moreover, in our previous studies [41,81] it has been shown that melatonin did not induce changes in hemodynamics, oxidative parameters, or NO-synthase activities in the control group. Thus, it might be justified to suppose that melatonin would not have an impact on the structure of the left heart in rats not afflicted by a pathologic process.

## 6. Conclusions

We conclude that the chronic administration of lactacystin represents a novel model of hypertension with collagenous rebuilding of the LV, convenient for testing antihypertensive drugs or agents exerting a cardiovascular benefit beyond blood pressure reduction.
